# Impact of non-pharmaceutical interventions during the COVID-19 pandemic on pathogens transmitted via food in the Netherlands

**DOI:** 10.1017/S0950268824000815

**Published:** 2024-10-22

**Authors:** Roan Pijnacker, Lapo Mughini-Gras, Linda Verhoef, Maaike van den Beld, Eelco Franz, Ingrid Friesema

**Affiliations:** 1 Centre for Infectious Disease Control, National Institute for Public Health and the Environment (RIVM), Bilthoven, The Netherlands; 2 Institute for Risk Assessment Sciences, Utrecht University, Utrecht, The Netherlands; 3 Office for Risk Assessment & Research, Netherlands Food and Consumer Product Safety Authority (NVWA), Utrecht, The Netherlands

**Keywords:** COVID-19, foodborne infections, non-pharmaceutical interventions, surveillance, public health

## Abstract

The COVID-19 pandemic impacted the transmission of many pathogens. The aim was to determine the effect of non-pharmaceutical interventions on the incidence of diseases transmitted via food. Weekly incidence rates for nine foodborne pathogens were collected from national surveillance registries. Weekly pathogen incidence during lockdown weeks of 2020 and 2021 were compared with corresponding weeks in 2015–2019. The same analyses were performed to determine the effect of self-defined expected impact levels of measures (low, intermediate and high). Eight out of 9 diseases showed a significant decrease in case number in 2020, except for listeriosis, which remained unchanged. The largest decrease was observed for rotavirus gastronteritis A (−81%), norovirus gastroenteritis (−78%), hepatitis A (−75%) and shigellosis (−72). In 2021, lower case numbers were observed for 6 out of 9 diseases compared with 2015-2019, with the largest decrease for shigellosis (−5/%) and hepatitis E (−47%). No significant change was observed for listeriosis, STEC infection and rotavirus gastroenteritis. Overall, measures with increased expected impact level did not result in a larger decrease in number of cases, except for Campylobacter, and norovirus and rotavirus gastroenteritis. Disease transmitted via food significantly decreased during the COVID-19 pandemic, with a more pronounced effect during 2020 than 2021.

## Introduction

On 11 March 2020, the World Health Organization announced the pandemic of coronavirus disease 2019 (COVID-19), caused by severe acute respiratory syndrome coronavirus 2 (SARS-CoV-2). In the Netherlands, extensive non-pharmaceutical interventions (NPIs) were taken to decrease the transmission of SARS-CoV-2, including local and international travel restrictions, lockdowns and stay-at-home recommendations, universal handwashing guidance, physical distancing, and face covering. These preventive measures did not only affect the spread of SARS-CoV-2, but also other infectious diseases. Most studies have focused on significant declines in other respiratory viruses, such as influenza, influenza-like illness, and pertussis, but decreases in several invasive diseases, vaccine preventable disease, and gastrointestinal diseases have also been reported [1–10]. Although the impact of NPIs was greatest for pathogens that (indirectly) spread from person to person, decreases have also been observed for pathogens associated with foodborne transmission.

Although the effect of NPIs on foodborne transmission might be less obvious, it could be related to measures such as closure of restaurants and increased hand hygiene. Likewise, there have been numerous reports on underdiagnosis and underreporting due to healthcare systems being under enormous pressure, as well as healthcare avoidance, that may have impacted the number of cases captured by surveillance [1, 5, 7–9]. Because the relative importance of foodborne transmission varies per pathogen, as well as the type of food products involved, differences in the impact NPIs had per pathogen are expected to vary. For example, previous studies found the impact to be largest on viral gastrointestinal infections, such as norovirus and rotavirus, compared with bacterial gastrointestinal infections such as *Salmonella* and *Campylobacter* [5, 9, 11, 12]. This is likely because the fraction of infections attributable to person-to-person transmission is much larger for norovirus and rotavirus than it is for *Salmonella* and *Campylobacter* [13].

The COVID-19 pandemic offers an opportunity to gain more insight into the drivers of gastrointestinal infections transmitted via food. The aim of this study was to determine the impact of the NPIs during COVID-19 pandemic on the incidence of bacterial and viral diseases that can be transmitted through food.

## Methods

### Data collection

Diseases were selected based on their ability to be transmitted via food and availability of national surveillance data during 2015–2021. These included five bacterial (campylobacteriosis, salmonellosis, shigellosis, Shiga toxin-producing *Escherichia coli* (STEC) infections, and listeriosis) and four viral diseases (hepatitis A, hepatitis E, norovirus, and rotavirus gastroenteritis).

Data on campylobacteriosis in the Netherlands were obtained from the Infectious Disease Surveillance Information System for Antibiotic Resistance (ISIS-AR), which collects data on antibiotic resistance from a large number of medical microbiology laboratories in the Netherlands. In 2021, it had an estimated nationwide coverage of 64%. Epidemiological data are limited to age, gender, date of sampling, and serotype. Data on salmonellosis are obtained via the national laboratory surveillance carried out by the National Institute for Public health and the Environment (RIVM), with an estimated coverage of 64% as well. Epidemiological data included age, gender, travelling abroad (reported in a subset of cases), date of sampling, and serotype.

Infections with *Shigella*, STEC, *Listeria monocytogenes*, and hepatitis A virus are notifiable in the Netherlands. Age, gender, report of travelling abroad, and date of illness onset and serotype (for *Shigella*) were extracted from the notifications. For STEC, *Listeria monocytogenes*, and hepatitis A virus, serotype information was obtained from the voluntary laboratory surveillance systems at the RIVM.

Data on hepatitis E, norovirus, and rotavirus were obtained from the Virological Laboratory Surveillance, consisting of approximately twenty virological laboratories that serve primary and/or secondary care, affiliated with the Dutch Working Group for Clinical Virology (NKWV) of the Dutch Association for Medical Microbiology (NVMM), that weekly report the aggregated number of detections of a large number of viral pathogens. These data do not contain information on gender, age, or travelling.

## Measures

In the Netherlands, the first measures to control the spread of SARS-CoV-2 were implemented in week 11 of 2020 and the last measures were lifted in week 15 of 2022. We constructed a timeline by week where measures were categorised a priori according to their self-defined expected impact on daily life (0 = no measure or very low impact; 1 = some restrictions apply, but estimated impact is low; 2 = moderate impact; 3 = high impact/lockdown in place). The level of the lockdown measure was used as basis for defining the impact extended with the impact of the other measures (see Supplementary Table S1 and Supplementary Figure S1). Three binary variables were constructed, one for each defined impact level, where one were the weeks with that impact level, and 0 were the same weeks in 2015–2019. During the COVID-19 lockdown period, various containment measures such as stay-at-home order, travel restrictions, and business closures were often implemented concurrently. Hence, multicollinearity between measures hampered our ability to study the effect of individual measures, and we could only study the combined effect of measures.

## Analyses

For each disease, we determined the association between the weekly disease incidence and the NPIs to control the spread of SARS-CoV-2. For this purpose, the incidence in the weeks of the measures during 2020 (week 11–52) and 2021 (week 1–52) were compared with the incidence in the same weeks during 2015–2019. Per disease, generalized linear models with Poisson distribution and log link function, or negative bionomical distribution in case of overdispersion, were used, with the weekly disease incidence as outcome and lockdown as binary dependent variable. Age (categorized into 0-4, 5-17, 18-39, 40-59, and 60+ years of age) and gender were always adjusted for. Data on population were included as offset variable to account for changes in demographics over the years and were obtained via Statistics Netherlands (www.cbs.nl). The same analyses were also performed by age groups and gender. Analyses by age groups were adjusted for gender and vice versa. Moreover, the analyses were repeated after exclusion of travel-related cases for diseases for which travel information was available and travel was reported as route of infection at least once during lockdown. These additional analyses were only possible for hepatitis A, listeriosis, salmonellosis, shigellosis, and STEC due to data availability. To determine whether there was a gradual decrease in number of cases with increasing impact of NPIs, a separate model was built for each of the impact score (i.e. low, moderate, and high) per disease. For six pathogens, separate models were used per subtypes to identify changes in number of cases by subtype: *Listeria monocytogenes* (serotypes 1/2a, 1/2b, 4b), *Campylobacter* (species *jejuni* and *coli*), STEC (serogroups O157, O26, and other), *Shigella* (species *sonnei, flexneri*), *Salmonella* (serotypes Enteritidis, Typhimurium, other), and hepatitis A virus (genotype I.A, I.B, III.A). For each of these analyses, separate models were made for 2020 and 2021, comparing the weeks in those years with the same weeks in 2015–2019. Furthermore, Pearson’s correlation coefficient was used to identify the lag with the strongest correlation between case count and the measures during 2020, which was the lag used for analyses. A lagged case count variable was entered in the model by shifting the values of the predictor variable by the number of weeks with the strongest correlation. The lag was restricted to a maximum of one week above the usual incubation period, with a maximum of four weeks (see Supplementary Table S2). Because there was a large international outbreak of hepatitis A among men who have sex with men (MSM) in 2017–2018 [14], cases related to this outbreak were excluded. Results are reported as exponentiated coefficients, subtracted by 1, with corresponding 95% confidence intervals (95% CI), which can be interpreted as the percentage increase or decrease in number of cases compared with 2015–2019. Analyses were performed in RStudio (version 2022.07.02).

## Results

**Figure 1.**
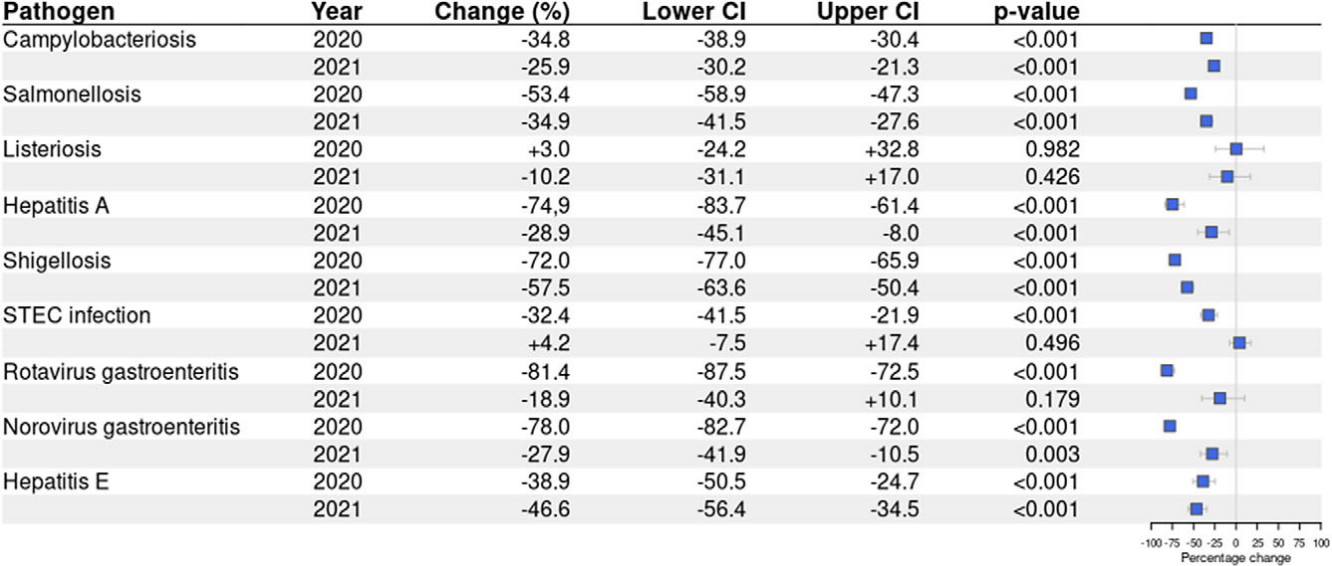
Changes in disease incidence during weeks with measures in 2020 (week 11-52) and 2021 (week 1-52) compared with the same weeks in 2015-2019, including both domestically acquired and travel related cases.

**Figure 2.**
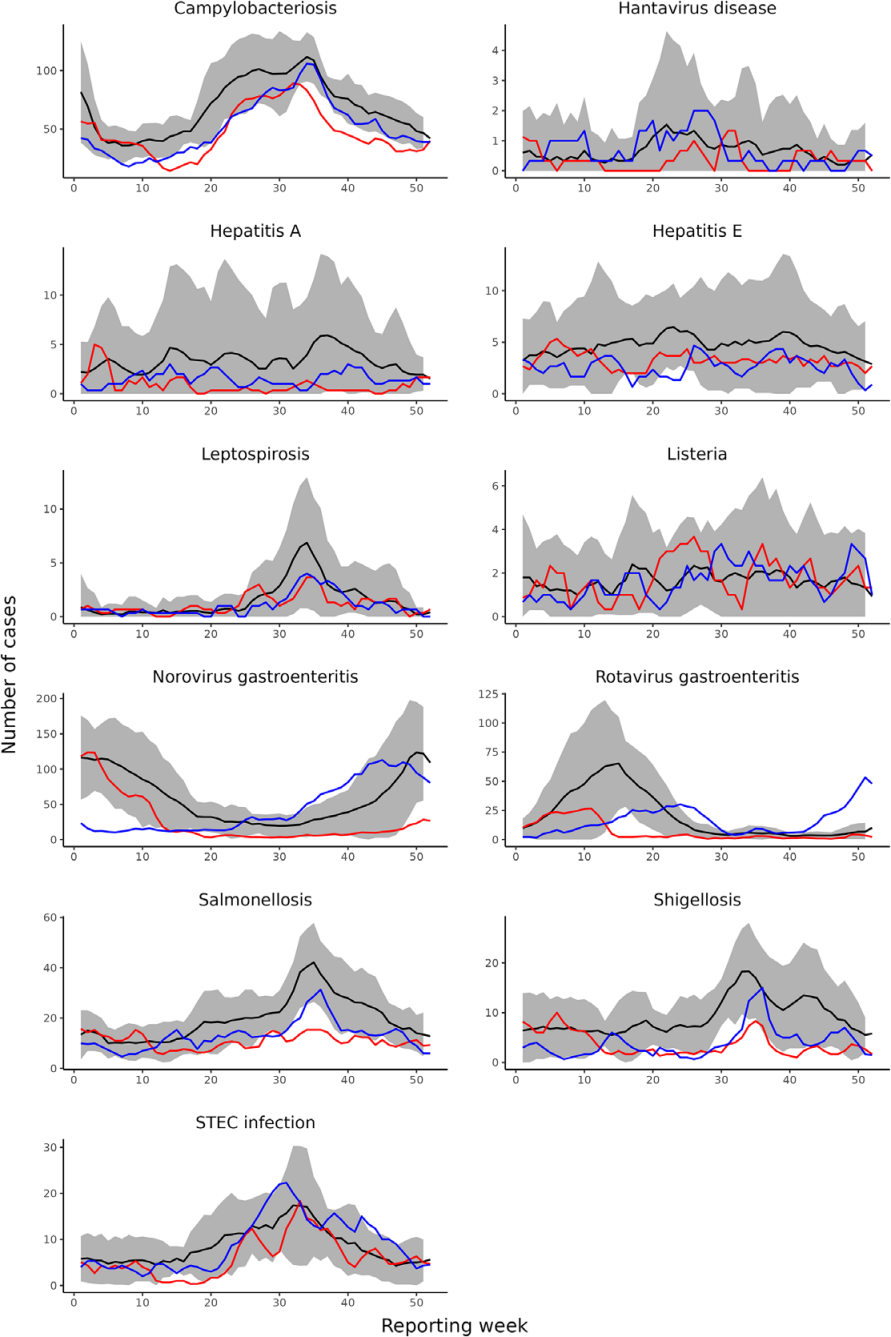
Weekly case numbers using three-week moving averages by disease in 2015-2019 (black line,including 95% confidence interval), 2020 (red line), and 2021 (blue line).

During the lockdown period in 2020, eight out of nine diseases showed a significant decrease in weekly disease incidence compared with 2015–2019 (Figures 1 and 2). The largest decrease was observed for rotavirus gastroenteritis (-81.4%, 95%CI: -87.5%, -72,5%), norovirus gastroenteritis (-78.0%, 95%CI: -82.7%, -72.0%), hepatitis A (-74.9%, 95%CI: -83.7%, -61.4%), and shigellosis (-72.0%, 95%CI: -77.0%, -65.9%). This was followed by salmonellosis (-53.4%, 95%CI: -58.9%, -47.3%), hepatitis E (-38.9%, 95%CI: -50.5%, -24.7%), campylobacteriosis (-34.8%, 95%CI: -38.9%, -30.4%), and STEC infection (-32.4%, 95%CI: -41.5%, -21.9%). The number of cases in 2020 only remained similar to 2015–2019 for listeriosis (+3.0%, 95%CI: -24.2%, +32.8%).

In 2021, the number of cases was significantly lower than 2015–2019 for six out of nine diseases, but case numbers were overall higher than in 2020. The largest decrease was observed for shigellosis (-57.5%, 95%CI: -63.6%, -50.4%), followed by hepatitis E (-46.6%, 95%CI: -56.4%, -34.5%), salmonellosis (-34.9%, 95%CI: -41.5%, -27.6%), hepatitis A (28.9%, 95%CI: -45.1%, -8.0%), norovirus gastroenteritis (-27.9%, 95%CI: -41.9%, -10.5%), and campylobacteriosis (-25.9%, 95%CI: -30.2%, -21.3%). STEC infection (+4.2%, 95%CI: -7.5%, +17.4%), listeriosis (-10.2%, 95%CI: -31.1%, +17.0%), and rotavirus gastroenteritis (-18.9%, 95%CI: -40.3%, +10.1%) showed no significant decrease in number of reported cases in 2021.

## Effect by age and gender

**Figure 3.**
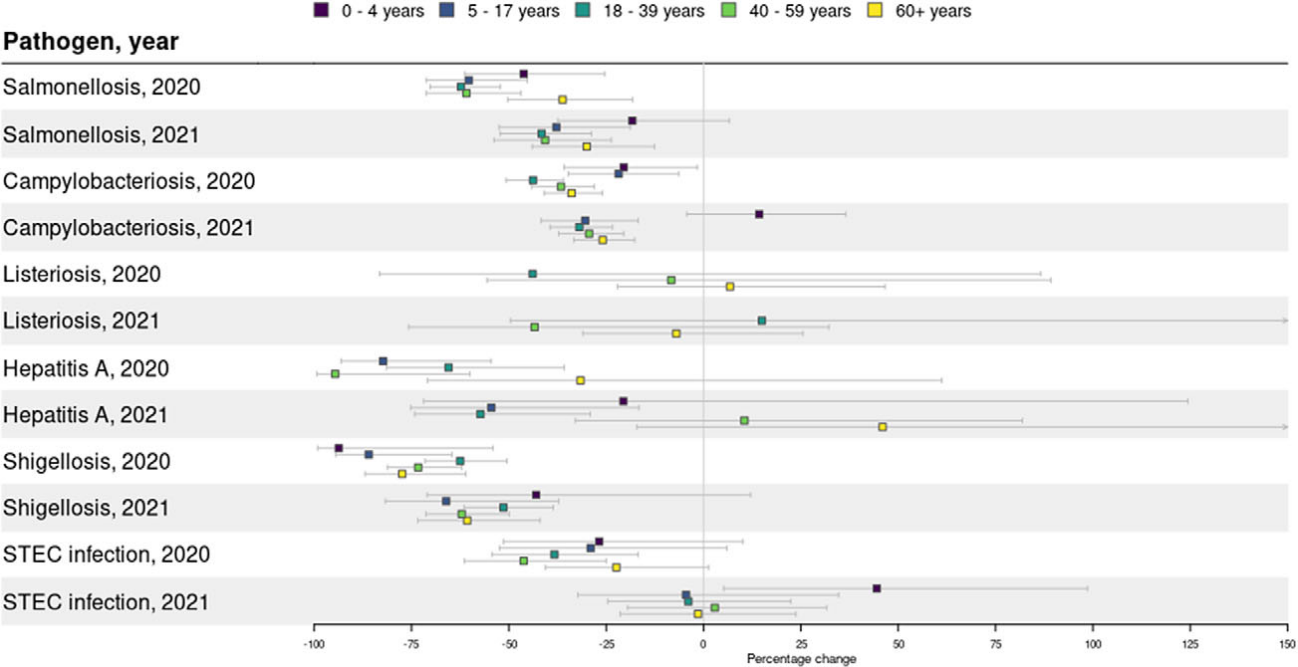
Changes in disease incidences during the lockdown weeks in 2020 (week 11-52) and 2021 (week 1-52) compared with the same weeks in 2015-2019, by age groups, including both domestically acquired and travel related cases.

Overall, the median percentage decrease in incidence over all diseases was less prominent with increasing age. A median decrease of 40.7% was observed in age group 0-4 years, 38.8% decrease in age groups 5-17, 37.8% decrease in age group 18-39 years, 36.6% decrease in age group 40-59 years, and 36.2% decrease in those 60+ years old (Figure 3). However, the age group 0-4 years decreased less compared to other age groups in 2021 for *Campylobacter* and STEC when comparing to case numbers in 2015–2019. Females generally had a larger decrease in disease incidence compared with males, with a median decrease of 35.6% over all diseases compared with 33.3% in males (Supplementary Figure S2). This was most pronounced for shigellosis, with a 85.5% decrease in 2020 (95%CI: -78.2%, -90.3%) and 73.0% decrease in 2021 (95%CI: -63.9%, -79.8%) in females, compared with a decrease of 60.9% (95%CI: -51,3, -68.7) and 45.0% (95%CI: -34.3%, -53.9%), respectively, in males.

## Effect on domestically acquired cases

**Figure 4.**
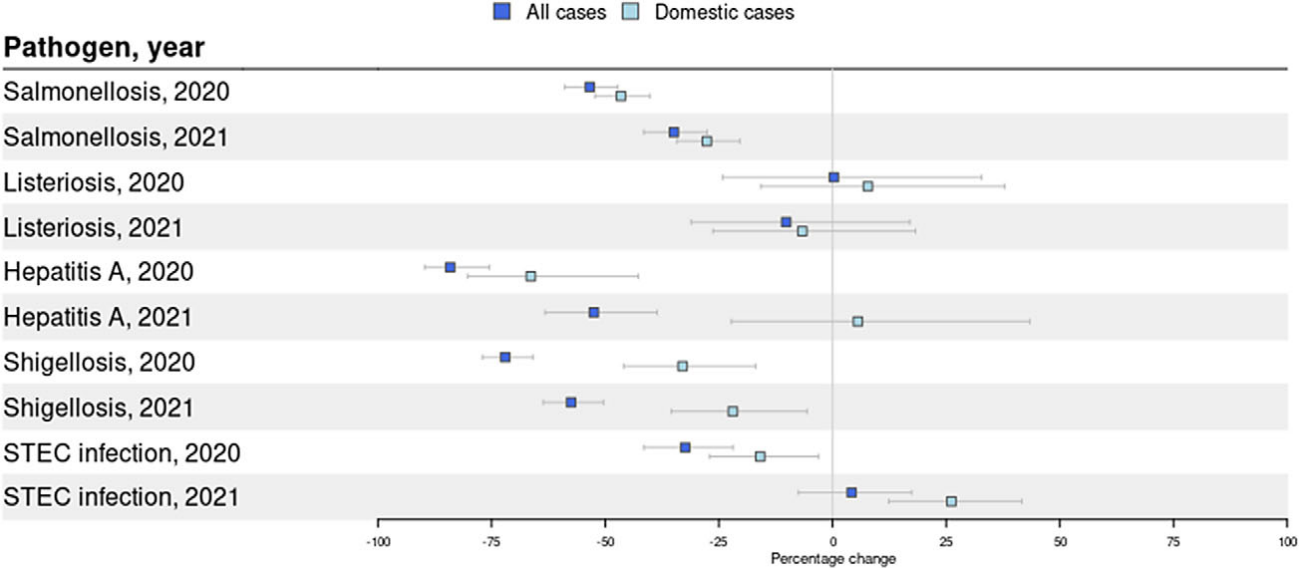
Changes in overall incidence and incidence of domestically acquired cases per disease during lockdown weeks in 2020 (week 11-52) and 2021 (week 1-52) compared to the same weeks in 2015-2019. The dark blue boxplots represents the percentage change in the overall disease incidence (travel and domestically acquired cases) and the light blue boxplots represents the percentage change in the disease incidence of domestically acquired cases.

To determine the effect that NPIs had on domestically acquired cases, travel-related cases were excluded from the analyses for five out of nine diseases for which information on travel was available. Overall, the decrease in total number of cases was larger than the decrease in domestically acquired cases only, although this difference was often not significant (Figure 4). Although the number of domestically acquired cases was still significantly lower in 2020 and 2021 compared with 2015-2019 for shigellosis, the decrease was significantly less pronounced.

## Effect of impact level of measures

**Figure 5.**
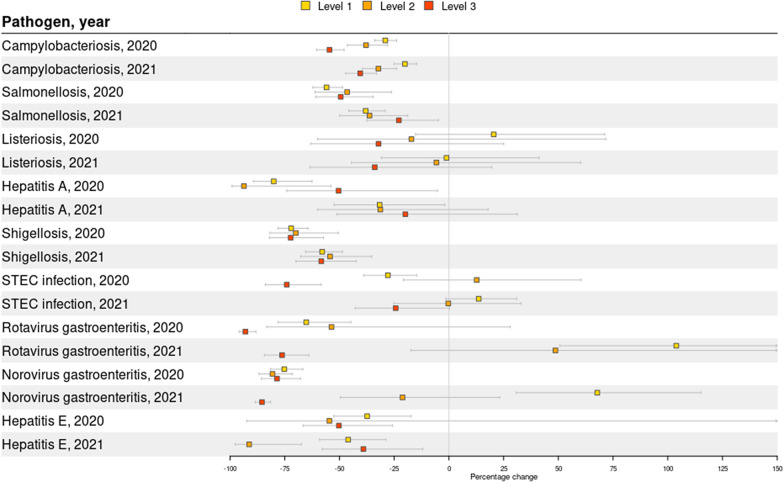
Changes in disease incidence per expected impact level in 2020 and 2021 compared to 2015-2019. Level 1 was defined as a mild impact and level 3 as a high impact (lockdown).

The expected effect of the measures on the number of cases was also assessed by levels of impact, with level 1 being mild expected impact and level 3 being high impact (i.e. lockdown). For campylobacteriosis, the number of cases in 2020 decreased -29.1% (95%CI: -23.9%, -33.9%) during level impact 1, -37.9% (95%CI: -28.0%, -46.4%) during level 2, and -54.6% (95%CI: -47.9%, 60.4%) during level 3 (Figure 5). In 2021, this was 20.1% (95%CI: -14.8%, -25.0%), 32.2% (95%CI: -24.0%, -39.6%), and 40.5% (95%CI: -33.0%, 47.2%), respectively. For rotavirus gastroenteritis, the number of cases increased +103.9% (95%CI: 50.7%, 175.9%) during level 1, +48.7% (95%CI: -17.4%, +167.5%) during level 2, and decreased -76.2% during level 3 (95%CI: -64.0%, -84.3%). For norovirus gastroenteritis, the number of cases increased +67.8% (95%CI: 30.8%, 115.2%) during level 1, and decreased -21.2% (95%CI: --49.6.4%, +23.2%) during level 2, and decreased -85.4% during level 3 (95%CI: -88.5%, -81.5%). Other diseases, however, had varying impact per level, without apparent larger effect of higher impact levels on the number of cases.

## Effect by subtype

For six pathogens, analysis was stratified by subtype. The number of *Shigella sonnei* cases had dropped significantly more in 2020 and 2021 than of S*higella flexneri* (Supplementary Figure S3). When excluding travel-related cases, there was no significant decrease in number of domestically acquired *Shigella flexneri* cases in 2020 and even a significant increase in 2021. Finally, for hepatitis A, the number of cases only significantly decreased for type I.A in 2020 and only for the overall number of cases with type I.A in 2021, but not for the domestically acquired cases. No significant differences were seen between the most common serotypes of *Campylobacter*, *Listeria monocytogenes, Salmonella*, and STEC.

## Discussion

During the COVID-19 pandemic, a continuum and variety of control measures were applied to prevent the spread of SARS-CoV-2. In addition to the intended effect – curbing the number of COVID-19 cases – it also affected a large number of other infectious diseases. Nine diseases that can be transmitted via the foodborne route were examined in the present study, including campylobacteriosis, hepatitis A, hepatitis E, listeriosis, norovirus gastroenteritis, rotavirus gastroenteritis, salmonellosis, shigellosis, and STEC infection. In 2020, the number of cases of all studied diseases except listeriosis had decreased. In 2021, the overall decrease in number of cases was less pronounced, with significantly lower case numbers for six out of nine diseases, including campylobacteriosis, hepatitis E, norovirus gastroenteritis, salmonellosis, and shigellosis. Overall, case numbers decreased most in the younger age groups.

The largest decrease in case numbers was observed for pathogens with a higher fraction of infections attributable to person-to-person transmission, including norovirus and rotavirus gastroenteritis, and *Shigella*, particularly during 2020, compared to pathogens mainly transmitted through food, such as *Campylobacter* and *Salmonella* [15]. We hypothesize that this could be because NPIs had differential impact depending on transmission route, mainly impacting person-to-person transmission due to increased emphasis on hand hygiene and physical distancing. Alternatively, travel restrictions, decreased dine-in options, and increased hygiene may have also contributed to decreased foodborne transmission. However, the reasons for changes in case numbers are likely to be multifactorial and may not reflect true changes in disease incidences. For example, we could not take into account changes in healthcare seeking behaviour and laboratory testing, which may have impacted the number of cases captured by surveillance [16]. Moreover, the impact may even be disease-specific, with a larger effect on disease with a milder course of the disease, such as norovirus for which patients may have refrained from seeking medical care, compared with campylobacteriosis and salmonellosis, which are more often associated with bloody stools. Findings in this study were largely consistent to previous studies reporting on the effect of NPIs on gastrointestinal and/or foodborne infections. For example, Germany reported a larger decrease in 2020 for rotavirus gastroenteritis, norovirus gastroenteritis, and shigellosis than for salmonellosis and campylobacteriosis, and similar findings were reported in Australia, England, Korea, Switzerland, and the United States [5, 9, 17–21]. Although most other studies have limited overlap in terms of foodborne infections included in our analyses, making comparison difficult, they generally also reported larger reductions for viral enteric diseases compared to bacterial enteric diseases [20, 22–25].

Among all age groups, the youngest (0-4 years) had the largest decrease in number of cases, which was also observed for a large range of notifiable diseases in Germany and only in the youngest age groups in England [5, 9]. Whether this is due to changes in healthcare utilization per age group or reflects true changes in number of cases is difficult to evidence. However, we hypothesize that cases were more likely to be diagnosed in the younger and older age groups compared to other age groups during the COVID-19 pandemic because of their vulnerability.

Listeriosis was the only disease with a stable number of cases during the COVID-19 pandemic compared to the years before. This suggests that the transmission route of *Listeria monocytogenes*, mainly pre-packaged, ready-to-eat food, stored and prepared without thorough heating at home, was not altered. An increase in underdiagnosis, underreporting, and healthcare avoidance may have had limited effect on listeriosis because of its severity almost always leading to hospitalization. Although similar findings were reported in Korea, Germany and the United States observed a significant decrease in the number of listeriosis cases in 2020; however, the number of listeriosis cases in the United States were back at pre-pandemic levels in 2021 [5, 10, 1 8, 26]. The reason for these differences between countries, however, is unknown.

After a substantial drop in the number of rotavirus cases in the Netherlands in 2020, the number of rotavirus cases did not significantly decrease in 2021 compared with pre-COVID-19 years. This was due to an early onset of the 2022 rotavirus season in October 2021, which was most likely the result of an accumulation of susceptible children that did not acquire a rotavirus infection during 2020 and 2021 rotavirus season. A similar phenomenon was observed for respiratory syncytial virus (RSV) in the Netherlands, for which a drop in antibody concentrations was observed during the COVID-19 pandemic, resulting in out-of-season RSV activity [27]. In Northern China, a rebound of rotavirus gastroenteritis was also observed after re-opening of schools in September 2020 [19].

Most of the reduction in shigellosis cases was due to a drop in travel-related cases. Subtype-specific analyses revealed that the number of domestically acquired *Shigella flexneri* cases was similar to pre-COVID-19 years in 2020 and even significantly higher in 2021. This was because the number of *Shigella flexneri* cases among MSM significantly increased in 2021 compared with pre-COVID-19 years [28]. This could also explain the larger decrease that was observed in females compared with males. The number of domestically acquired *Shigella sonnei* infections, however, decreased significantly during both pandemic years. For hepatitis A, a significant drop in the overall number of hepatitis A cases was observed, but not for the number of domestic cases. This could be because of two outbreaks in 2021: one with genotype I.A (14 cases) and one with I.B (7 cases). Because cases had not travelled and were geographically spread, the source was most likely a food product, but could not be confirmed.

Analyses on the different impact levels of measures showed that only the number of campylobacteriosis cases gradually decreased with an increasing impact of NPIs. A potential reason could be that the impact of the measures leads to shifts in transmission possibilities of the pathogen. For example, full closure of restaurants during the highest impact level facilitates spread of foodborne pathogens through take-away or delivery services. However, a similar effect would then be expected for *Salmonella*, which has relatively similar transmission routes compared with *Campylobacter.* Alternatively, the period of measures with the highest impact was generally during time periods when the incidence for most of the pathogens included in this study is usually low. Indeed, measures with the highest impact level were mainly in place during January to May, while the number of cases for many of the included diseases is highest during summer, including campylobacteriosis, shigellosis, and STEC infection. This likely also explains why the number of rotavirus and norovirus gastroenteritis cases only dropped significantly during measures with the highest impact level in 2021, because these measures were during the months of the usual norovirus and rotavirus season.

The effect of NPIs on case numbers was generally larger in 2020 than in 2021. A potential explanation could be that the compliance of the Dutch general population to the NPIs decreased throughout the course of the pandemic, or because there were different types or less stringent NPI’s [29]. Furthermore, healthcare avoidance, underdiagnosis, and underreporting may also have been more prevalent in 2020 compared to 2021, although this is difficult to [16]. Preliminary data from the United States report similar findings, with the incidence of foodborne diseases including campylobacteriosis, listeriosis, shigellosis, and STEC infection, increasing in 2021 compared with 2020, and sometimes even stabilizing or increasing compared with pre-COVID-19 years [18, 26]. However, they hypothesize that this may have been due to lifting of some of the pandemic control measures, while our analyses only focuses on weeks in 2021 where control measures were in place.

Our study has several limitations. First, we cannot infer causality of the impact of NPIs on pathogen circulation from the surveillance data. Based on our analyses, it is not possible to distinguish between the effect of NPIs and the effect of healthcare avoidance, underdiagnoses, and/or underreporting due to healthcare being overwhelmed and concerns around infection risks during healthcare visits. Second, most of the NPIs were implemented at the same time, making it impossible to separate the effect of, for example, restaurant closures from social distancing. Therefore, it was not possible to estimate measure-specific effects.

Concluding, the COVID-19 pandemic has had a major impact on foodborne infections in the Netherlands. However, disentangling the impact specific NPIs had on the effect of foodborne disease circulation from the effect of, for example, healthcare avoidance and testing strategies is challenging. Overall, the effect of the COVID-19 pandemic seems to have been largest for diseases with a lower fraction of cases being attributable to foodborne transmission. Moreover, case numbers were more heavily impacted in 2020 compared with 2021, with an upsurge in cases for rotavirus gastroenteritis at the end of 2021. The latter also highlights the importance of close monitoring of foodborne diseases as they may follow an untraditional seasonal pattern in coming years due to an accumulation of susceptible persons.

## Supporting information

Pijnacker et al. supplementary material Pijnacker et al. supplementary material

## Data Availability

The data that support the findings of this study are available on request.

## Supplementary material

For supplementary material accompanying this paper visit http://doi.org/10.1017/S0950268824000815. click here to view supplementary material
