# Supplementary material for: Impact of non-pharmaceutical interventions during the COVID-19 pandemic on pathogens transmitted via food in the Netherlands
Source: Epidemiol Infect. 2024 Oct 22;152:e130. doi: 10.1017/S0950268824000815 (PMC11502420; doi:10.1017/S0950268824000815)
Supplement: Pijnacker et al. supplementary material [file S0950268824000815sup001.docx]

Supplementary material for Epidemiology & Infection

Title: Impact of non-pharmaceutical interventions during the COVID-19 pandemic on pathogens transmitted via food in the Netherlands

Roan Pijnacker, Lapo Mughini-Gras, Linda Verhoef, Maaike van den Beld, Eelco Franz, Ingrid Friesema

**Measures**

In the Netherlands, the first measures to control the spread of SARS-CoV-2 were implemented mid-March 2020 (week 11) and the last measures were lifted mid-April 2022 (week 15). A timeline with measures per week was constructed, grouping the measures in level and adding a weight to the impact of the applicable measure: 0 (green) = no measure or very low impact; 1 (yellow) = some restrictions apply, but estimated impact is low; 2 (orange) = impact of the measure is estimated to be moderate; 3 (red) = impact of the measure is estimated to be high. Table 1 shows the levels and weights. An overall variable was constructed, using the level of impact of the lockdown measure as basis extended with the impact of the other measures.

In Figure S1 a schematic overview is given of the different groups of measures per week for the period week 1 2020 up to week 26 2022. First measures were taken in week 11 of 2020 and were in most cases immediately with the highest impact on daily life. Strictest measures applied in two periods, namely in the weeks 13-22 of 2020, and from week 51 (2020) up to week 16 (2021). The overall variable with the level of impact as used in the study is given in the first row.

**Supplementary Table S1**. Weights assigned to the groups of control measures taken during the pandemic

|  | Severe impact (3) | Moderate impact (2) | Low impact (1) | Normal (0) |
| --- | --- | --- | --- | --- |
| Lockdown | lockdown | stay at home advice | avoid large groups | (almost) no restrictions |
| Distancing | mandatory | advice |  | no restrictions |
| Use of masks | standard | in many places | public transportation only | no restrictions |
| Travelling abroad | only if necessary | under strict rules | under limited rules | (almost) no restrictions |
| Public transport | only if necessary | avoid rush hours | allowed, with rules (e.g. masks) | (almost) no restrictions |
| Gatherings | <50 participants | 50-<100 participants | 100+ participants | (almost) no restrictions |
| Visitors at home | 1-2 visitors | 3-4 visitors | 6 visitors | no restrictions |
| Education^*^ | closed | limited lessons at school | 50% lessons at school | (almost) no restrictions |
| Work from home | standard at home | to office allowed for meetings/if necessary | at least 50% at home | no restrictions |
| Contact professions | closed | only (para)medics | limited opening hours/use of masks mandatory | open (including health checks/registration of clients) |
| Sex workers | closed | limited opening hours (untill 5 or 6 PM) | opening hours limited from 5 AM to 10 PM | no restrictions |
| Catering industry / restaurants | closed/only takeaway | only terrace/very limited hours | partly open/under restrictions | open (including with corona-app) |
| Non-essential shops  Libraries | closed | takeaway/by appointment | limited opening hours | open (incl distancing/5 AM-10 PM) |
| Cultural institions  Museums/monuments | closed | severe restrictions/limited hours | open, but with restrictions | open (including with corona-app) |
| Sport | closed | very limited: children only/outdoor/2 persons per room/etc | sport is allowed, competion is not | competition also allowed (including no spectators allowed) |

^*^ 4 groups within Education: school care/daycare, primary education, secondary education, higher education

**Supplementary Figure S1**. Control measures by week and year, week 1 of 2020 to week 52 of 2021. Weeks without control measures are shown in green, low impact measures in yellow, intermediate impact measures in orange and severe impact measures in red.

**Supplementary Table S2**. Pearson correlation coefficient of the correlation between the lockdown measures in 2020 and the weekly number of cases. The lag was restricted to a maximum of one week above the usual incubation period, with a maximum of four weeks. The lag with the highest correlation was used for analyses and is highlighted in green.

|  | **Lag in weeks** | | | | |
| --- | --- | --- | --- | --- | --- |
| **Disease** | 0 | -1 | -2 | -3 | -4 |
| Campylobacteriosis | -0,2444 | -0,2460 | -0,2444 | -0,2457 | -0,2452 |
| Salmonellosis | -0,3194 | -0,3132 | -0,3084 | -0,3062 | -0,3142 |
| Listeriosis | -0,0328 | -0,0522 | -0,0386 | -0,0250 | -0,0124 |
| Hepatitis A infection | -0,2582 | -0,2678 | -0,2673 | -0,2690 | -0,2738 |
| Shigellosis | -0,4187 | -0,4139 | -0,4078 | -0,4053 | -0,4013 |
| STEC infection | -0,1278 | -0,1220 | -0,1294 | -0,1288 | -0,1346 |
| Rotavirus gastroenteritis | -0,1218 | -0,0966 | -0,0814 | -0,0716 | -0,0645 |
| Norovirus gastroenteritis | -0,3229 | -0,3102 | -0,3086 | -0,3085 | -0,2983 |
| Hepatitis E infection | -0,1333 | -0,1280 | -0,1146 | -0,1003 | -0,0975 |


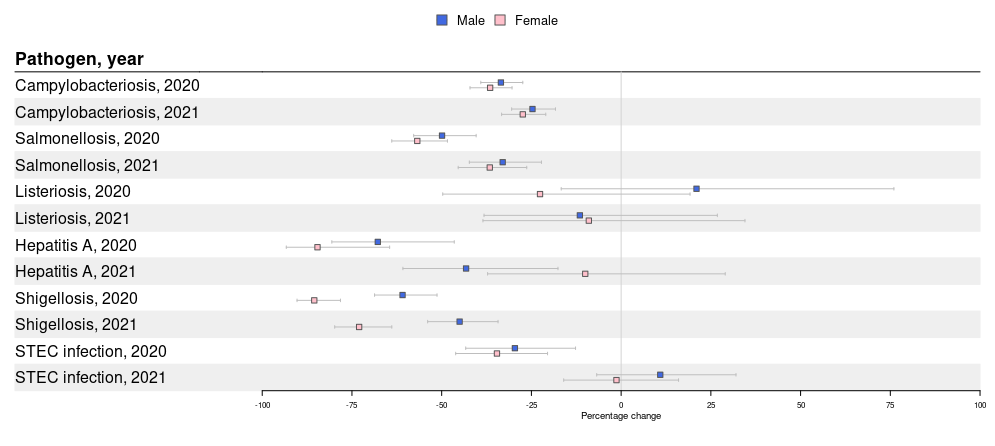


**Supplementary Figure S2**. Changes in disease incidence by gender for campylobacteriosis, salmonellosis, listeriosis, hepatitis A, shigellosis and STEC infection in 2020 and 2021 compared to 2015-2019. Data on gender was not available for Hepatitis E, rotavirus gastroenteritis and norovirus gastroenteritis.

**
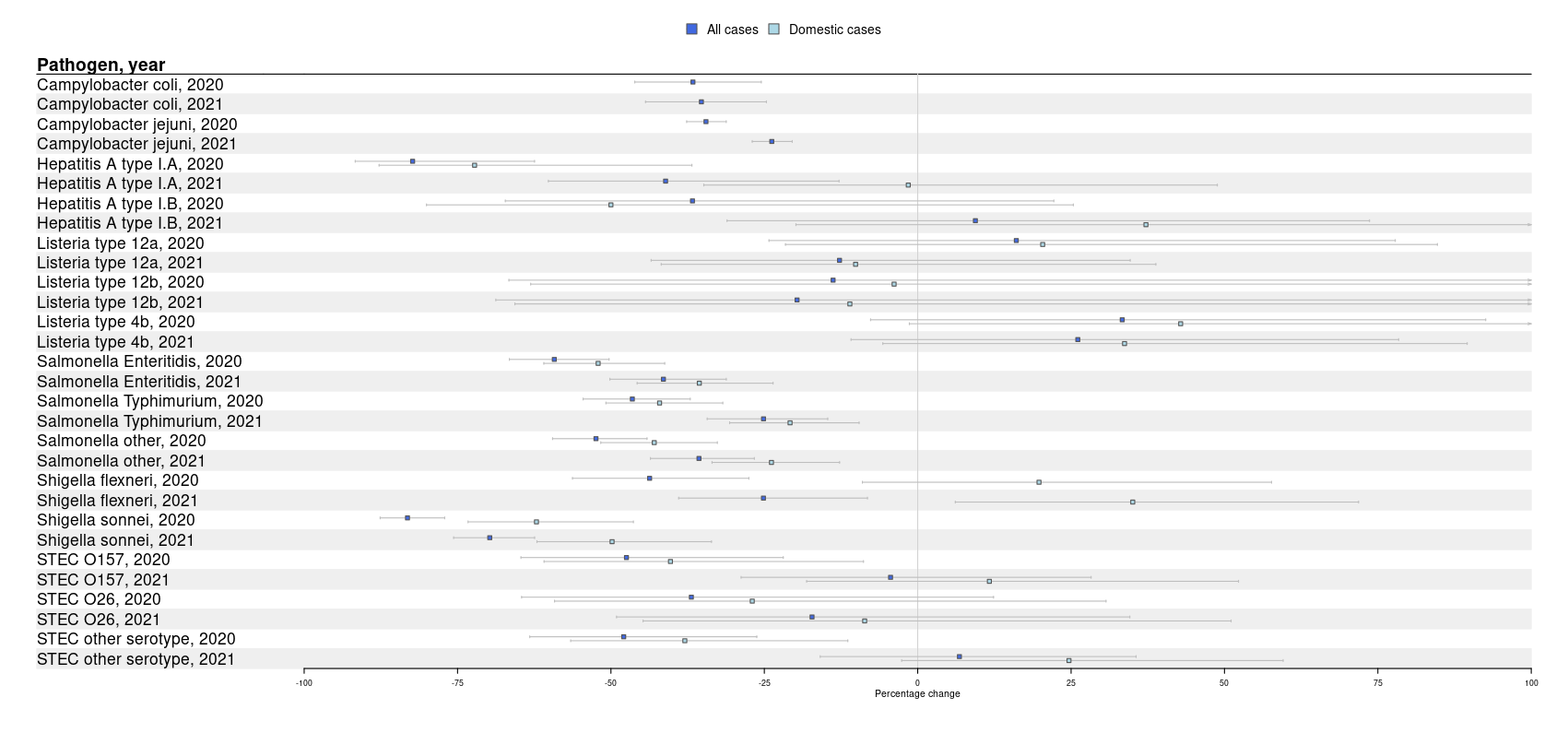
**

**Supplementary Figure S3**. Changes in disease incidence per serotype of Campylobacter, Hepatitis A, Listeria, Salmonella, *Shigella* and STEC in 2020 and 2021 compared to 2015-2019, stratified by all cases (domestically acquired and travel-related cases) and domestically acquired cases only. Data on travel was not available for *Campylobacter*.
